# Supplementary material for: Identification of photoperiod-induced specific miRNAs in the adrenal glands of Sunite sheep (Ovis aries)
Source: Front Vet Sci. 2022 Jul 22;9:888207. doi: 10.3389/fvets.2022.888207 (PMC9354845; doi:10.3389/fvets.2022.888207)
Supplement: Supplementary file 1 [file Data_Sheet_1.ZIP › Supplementary file/miRDeep2 file.zip!/miRDeep2 file/Novel_Structure/SPLP42A1/Novel_629.pdf]

Provisional ID	:	NC_019461.2_9772
Score total	:	2.3
Score for star read(s)	:	-1.3
Score for read counts	:	0
Score for mfe	:	2
Score for randfold	:	1.6
Score for cons. seed	:	
Total read count	:	24
Mature read count	:	23
Loop read count	:	1
Star read count	:	0

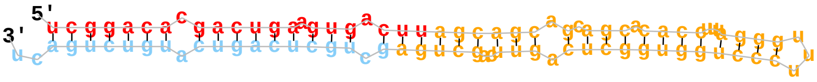

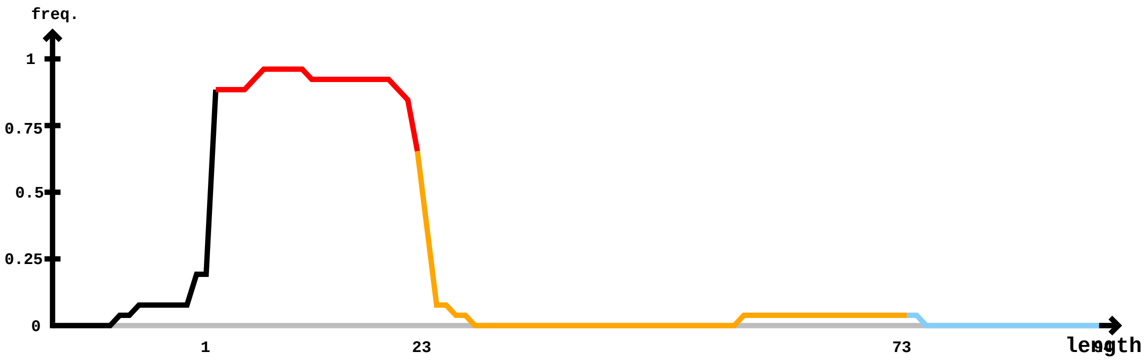

Mature **Star**

```
5'- uggggucgcacaaagucggacacgacugaagugacuuagcagcagcagcacaccuuuaggguuucccugguggcucaguucagcugagcugcucagucaugucugacuc -3'   exp
    ............((((((((.((((((.(((.(((((((((.(.(((.((((...((((...)))))))))))).))).)))..)))))).))))))))).)))))))).   reads   mm   sample
    .....ucgcacaGagucggacacga....................................................................................   1       1    seq
    .......gcacaGagucggacacgacugaaguga...........................................................................   1       1    seq
    .............aguUggacacgacugaagugacu.........................................................................   3       1    seq
    ...............ucggaUacgacugaagugac..........................................................................   1       1    seq
    ...............ucggacacgacugaagugacu.........................................................................   1       0    seq
    ...............ucggacGcgacugaagugacu.........................................................................   1       1    seq
    ...............ucggaUacgacugaagugacuu........................................................................   14      1    seq
    ...............ucggCcacgacugaagugacuu........................................................................   1       1    seq
    ...................acCgacugaagugacuuagc......................................................................   1       1    seq
    ....................cacgacugaagugacuuGgcag...................................................................   1       1    seq
    ......................................................................uggcucaguucagcugagc....................   1       0    seq
```
